# Liver and Intestinal Fatty Acid Binding Proteins Are Not Critical for Perfluorooctanesulfonate (PFOS) Tissue Distribution and Elimination in Mice

**DOI:** 10.1021/acs.chemrestox.5c00199

**Published:** 2025-08-19

**Authors:** Seyed Mohamad Sadegh Modaresi, Jitka Becanova, Simon Vojta, Sangwoo Ryu, Emily M. Kaye, Juliana Agudelo, Anastasia Diolintzi, Olga Skende, Judith Storch, Fabian C. Fischer, Angela Slitt

**Affiliations:** † Department of Biomedical and Pharmaceutical Sciences, 4260 University of Rhode Island, Kingston, Rhode Island 02881, United States; ‡ Graduate School of Oceanography, 4260 University of Rhode Island, Kingston, Rhode Island 02881, United States; § Department of Nutritional Sciences, 242612 Rutgers University, New Brunswick, New Jersey 08901, United States; ∥ Harvard John A. Paulson School of Engineering and Applied Sciences, Harvard University, Cambridge, Massachusetts 02138, United States

## Abstract

Perfluorooctanesulfonate (PFOS) is a persistent environmental pollutant in the per- and polyfluoroalkyl substances (PFAS) class, known to accumulate in the liver and trigger hepatotoxicity. While *in vitro* studies suggested that fatty acid-binding proteins (FABPs) drive the hepatic accumulation of PFAS, *in vivo* evidence is entirely lacking. Using wild-type and mice with global deletion of liver-type and intestine-type FABP (L-FABP^–/–^, I-FABP^–/–^), we measured PFOS toxicokinetics by administering single oral doses (0.1, 0.5, and 5 mg/kg) and tracking blood and excreta levels for 65 days. PFOS levels in various tissues were measured at test end. Additionally, we measured PFAS binding to liver tissues from wild-type and FABP knockout mice. Contrary to previous *in vitro* findings, FABP deletion did not significantly alter PFOS blood concentrations, tissue distribution, or elimination rates. Elimination half-lives, clearances, and volumes of distribution were consistent across genotypes, suggesting that neither L-FABP nor I-FABP are critical drivers for PFOS *in vivo* toxicokinetics. *In vitro* binding assays showed similar liver partition coefficients between wild-type and knockout livers for 15 of 19 PFAS, with small differences for some sulfonamides and fluorotelomer sulfonates. These results challenge the presumed role of L-FABP and/or I-FABP in PFAS toxicokinetics, highlighting the need to explore alternative toxicokinetic mechanisms—such as phospholipid binding and transporter-mediated uptake—driving PFAS distribution and elimination.

## Graphical abstract

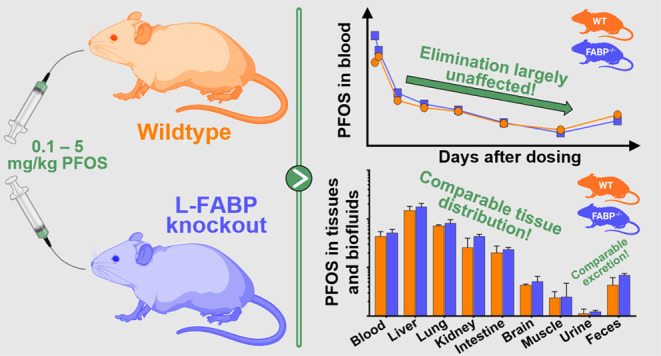

## 1 Introduction

Per- and polyfluoroalkyl substances (PFAS) are a class of over 15,000 man-made fluorochemicals found in a wide range of consumer and industrial products.^1,2^ A health concern for PFAS as a chemical class is that many PFAS are resistant to breakdown and biotransformation,^3^ which results in bioaccumulation in living organisms and detection in 99% of the general US population.^4^ Chronic exposure to certain PFAS, such as perfluorooctanesulfonic acid (PFOS), is associated with adverse health outcomes, such as decreased response to vaccination,^5,6^ hepatotoxicity,^7^ dyslipidemia,^8,9^ and testicular cancer.^10^ Containing eight fluorinated carbons in its carbon chain, PFOS is a hydrophobic PFAS with observed elimination half-lives in humans in the range of 4–6 years.^11^ PFOS is readily absorbed following oral exposure^11^ and is assumed to undergo enterohepatic circulation.^12^ After absorption, PFOS preferentially distributes to the liver,^13–15^ however, the mechanisms dictating PFOS liver accumulation and retarded elimination are poorly understood. *In vitro studies* have shown that fatty acid-binding proteins (FABPs) bind PFOS, and this has led to the suggestion that FABPs, particularly liver-type fatty acid binding protein (L-FABP; FABP1), which is abundant in the liver, drive PFOS liver accumulation.^16–18^ However, *in vivo* evidence is lacking, and the focus of the present work is to address this gap.

Because the chemical structure of PFOS and other PFAS resembles fatty acids, it has been postulated that PFOS bioaccumulation is facilitated by cellular mechanisms that dictate fatty acid uptake into the cell,^19^ as well as their intracellular binding.^20^ L-FABP is enriched in liver, representing as much as 5% of the total cytosolic protein. It binds long-chain fatty acids with high affinity,^21^ and has been suggested to be a key binding protein that mediates PFOS accumulation and retention in liver.^22^ PFOS binding to intestinal fatty acid binding protein (I-FABP; FABP2), which is exclusively present in intestinal epithelial cells, could be an additional important mediator of PFOS toxicokinetics. L-FABP and I-FABP have similar tertiary structures, yet PFAS binding to I-FABP has not been explored. Considering the high affinity of I-FABP to bind the fatty acids produced by hydrolysis of dietary lipid and its role in the uptake of fatty acids from the lumen of the intestine,^23^ we hypothesized herein that both L-FABP and I-FABP could be important proteins that drive PFOS toxicokinetics.

To date, research on the binding of PFAS to FABPs has primarily consisted of *in vitro* and *in silico* studies, largely using isolated FABP protein in binding assays or FABP-overexpression in *in vitro* cells.^18,24–28^ Dissociation constants (*K*_d_) for binding to L-FABP have been reported to range from 38 to 879 μmol/L for a list of perfluorocarboxylates and sulfonates, with PFOS exhibiting the strongest association among the PFAS tested.^29^ Similar trends were observed in other binding and molecular docking studies.^17,18,25^ Comparisons with *K*_d_ values for serum proteins like albumin indicate that PFAS binding to FABP is comparably strong.^22^ However, the contribution of L-FABP to PFOS tissue binding, distribution, and elimination in the presence of other relevant macromolecules (i.e., other proteins and phospholipids) in a complex *in vivo* system under physiological conditions has not been evaluated.

The present study was undertaken to examine, for the first time, whether binding to FABPs is a critical cellular mechanism for PFAS tissue distribution and elimination *in vivo*. The role of L-FABP or I-FABP binding in the presence of other physiologically relevant toxicokinetic processes *in vivo* was examined in mice that lack L-FABP or I-FABP expression in all tissues (i.e., global deletion of L-FABP^–/–^ or I-FABP^–/–^). Additionally, since L-FABP is highly expressed in both liver and intestine, conditional knockout mice lacking L-FABP specifically in intestine (L-FABPint^–/–^) or liver (L-FABPliv^–/–^) were examined. Mice were administered a single oral dose of PFOS and then blood, urine, and fecal concentrations were measured over the course of 65 days. PFOS concentrations were quantified in liver, kidney, lung, intestine, brain, and muscle tissue. Measured elimination half-lives and tissue distribution patterns in FABP knockout mice were compared to wild-type (WT) control mice with normal physiological levels of FABPs. Finally, we discuss our findings in the context of the relevance of FABPs in the toxicokinetics of PFAS in humans.

## 2 Materials and Methods

### 2.1 Chemicals

PFOS was purchased from Sigma-Aldrich as Heptadecafluorooctanesulfonic acid potassium salt (CAS: #2795-39-3, Catalog: #89374, ≥98.0% purity, ∼70% L-PFOS/∼30% Br-PFOS). For C18 fiber binding experiments, the PFAC-24PAR mixture from Wellington Laboratories was used, which includes carboxylates, sulfonates, fluorotelomers, and sulfonamides. Stable isotope-labeled internal standards for all study PFAS were purchased from Wellington Laboratories. roQ QuEChERS extraction packet kits and roQ QuEChERS dSPE kits were purchased from Phenomenex. Other chemicals and solvents, if not specified, were obtained from Sigma-Aldrich or Thermo Fisher Scientific.

### 2.2 Laboratory Animals and Husbandry

All animal protocols were reviewed and approved by the University of Rhode Island (URI) Institutional Animal Care and Use Committee (IACUC). The global deletions of L-FABP, which is normally highly expressed in both liver and intestine, and of I-FABP, normally expressed only in intestine, were verified by proteomics homogenate (Figure S1) and via Western blot using authentic purified proteins.^30,31^ Wild-type C57BL/6J served as controls. No compensatory changes in L-FABP levels were observed in intestine or liver of the I-FABP null mice, and no changes in I-FABP were found in the L-FABP null mice.^30,31^ Additionally, tissue-specific deletion in the intestine-specific L-FABP knockout (L-FABPint^–/–^) and liver-specific L-FABP knockout (L-FABPliv^–/–^) were also verified by proteomics (Figure S1 of the Supporting Information) and via Western blottin.^32^ Floxed L-FABP (L-FABP^fl/fl^) served as controls for the tissue-specific knockouts, with all mice on a C57BL/6J background. Again, no compensatory changes in either L-FABP or I-FABP were found in either tissue in the conditional knockout mice.^32^ Moreover, no compensatory changes were observed at the mRNA and/or protein level for FABP family members that are not normally expressed in intestine or liver, including FABP3 (muscle/heart FABP3), FABP4 (adipose FABP), FABP5 (skin FABP), FABP6 (ileal bile acid binding protein), and FABP7 (brain FABP).^31^ The mice (5–6 months old) were housed under a controlled temperature with relative humidity (30–70%) and 12-h light/dark lighting. Before and during the experiment, standard rodent food (Harlan Teklad Extruded Global Diet, 2020X) and water were provided ad libitum.

### 2.3 Single Dose Toxicokinetic Studies

PFOS was dissolved in 0.5% Tween 20 vehicle. Previous studies have shown that 0.5% Tween 20 does not affect pharmacokinetics.^33^ To confirm the absence of background contamination, we measured PFOS levels in tissues from WT mice treated with the vehicle and found them to be below the limit of detection. The overall goal of this study was to derive key toxicokinetic parameters typically obtained from a single-dose pharmacokinetic study. The study design used herein (i.e., single-dose over time) is consistent with numerous publications that have investigated ADME mechanisms using knockout mice and is widely accepted in the ADME field.^34,35^ To align with the FDA Modernization Act 2.0, which advocates for reducing animal use in testing, vehicle-treated mice were not included, as their inclusion is not standard for single-dose kinetic studies and is not essential for comparing genotypes. Initially and in order to test feasibility, a single dose of PFOS (5 mg/kg; 10 mL/kg) was administered by oral gavage to male wild-type mice (WT, *n* = 3), as well as male mice lacking L-FABP in the intestines (L-FABPint^–/–^, *n* = 3) or liver (L-FABPliv^–/–^, *n* = 3). These conditional knockouts were selected for initial screening to assess whether tissue-specific deletion of L-FABP produced notable changes in PFOS toxicokinetics. As no pronounced differences were observed, subsequent experiments focused on global knockout models of L-FABP (L-FABP^–/–^) and I-FABP (I-FABP^–/–^), which were tested at 0.1 and 0.5 mg/kg to evaluate systemic effects of FABP deletion under both environmentally relevant and higher-dose exposure conditions (*n* = 3–5). The selected doses and study design were based on prior pharmacokinetic studies evaluating PFOS kinetics in mice^11,36,37^ and were chosen to ensure PFOS could be reliably detected in small blood samples over 60 days while minimizing the number of animals used. The highest dose of 5 mg/kg was expected to be below the threshold for increased liver weights.^38,39^ After observing no significant toxicokinetic differences between genotypes at 5 mg/kg, 0.1 mg/kg and 0.5 mg/kg were included to assess potential effects at lower exposures. These doses fall within the range of previous PFAS pharmacokinetic studies in rodents, including those investigating PFOA and PFHxS.^36,37,40^ Additionally, the selected doses were expected to span both subsaturating and near-saturating conditions for PFOS-FABP binding. Based on the reported FABP dissociation constant (*K*_D_ = 0.18 μM)^16^ and the experimental unbound fraction of PFOS in mouse liver,^41^ free liver concentrations of PFOS at 0.1 mg/kg and 0.5 mg/kg were predicted to be ≈20 times below *K*_D_, whereas *K*_D_ was expected to be approached or exceeded at 5 mg/kg. This dose selection enabled the assessment of FABP’s role in PFOS toxicokinetics under both nonsaturating and potentially FABP-saturating conditions.

### 2.4 Sample Collection and PFOS Extraction

Body weight, food consumption, and tissues, serum, and liver weights were measured over 65 days postdosing. Blood, urine, and fecal samples were collected at 1, 2, 7, 14, 23, 35, 50, and 65 days postadministration for the 5 mg/kg group and at 1, 2, 7, 14, 24, 38, and 60 days postadministration for the 0.5 and 0.1 mg/kg groups. Urine collection was subject to variability, as some mice did not urinate within the allotted time in metabolism cages. This limitation was accounted for in data interpretation. PFOS was extracted using Phenomenex roQ QuEChERS kits, following the manufacturer’s instructions with slight modifications.^42^ Detailed procedures for sample collection and PFOS extraction are provided in Sections S1 and S2 of the Supporting Information.

### 2.5 Solid-Phase Microextraction Binding Assay

The binding of 24 PFAS to mouse blood and liver tissues was assessed using a previously developed C18 fiber-based solid-phase microextraction (SPME) technique. The SPME method was developed previously^20^ using purified serum proteins (albumin, γ-globulin) and demonstrated excellent recovery (≥75%), reproducibility across experiments (RSD ≤ 11%), equilibrium establishment within 48 h, and agreement with literature partition coefficients.^20^ In brief, C18 fibers (520 nL coating volume, Supelco #57281-U) were preconditioned by sequential incubation in methanol and deionized water prior to experiments. Liver tissues were homogenized by mechanical disruption using a Bullet Blender with five 4.8 mm stainless steel beads (Next Advance), precleaned sequentially with Milli-Q water, 0.4 M hydrochloric acid, 1% ammonium hydroxide in methanol, and methanol. Four milliliters of phosphate-buffered saline (PBS, pH 7.4) were added to each tube prior to homogenization. Preconditioned fibers were transferred into tubes containing either liver homogenates or pure PBS, both spiked with the PFAC-24PAR PFAS mixture at 400 ng/L (*n* = 4 each). Fibers were fully immersed and incubated at 37 °C with horizontal shaking for 48 h. After incubation, fibers were transferred into methanol solutions prespiked with a mass-labeled PFAS mix (Wellington Laboratories) for overnight extraction (>12 h). PBS solutions were diluted 1:1 with methanol and spiked with internal standards. All fiber extracts and PBS solutions were vortexed prior to HPLC-MS/MS analysis. The difference in PFAS mass extracted by the SPME fiber between PBS and liver homogenate samples was used to determine the fraction of PFAS bound to liver tissue. From these measurements, liver partition coefficients (*K*_liver_) were calculated to quantify PFAS affinity for tissue components.

### 2.6 Measurement of PFAS by LC-MS

PFOS concentrations in biofluids and tissues were quantified in target mode using an ultraperformance liquid chromatography system (UPLC) coupled with a triple quadrupole 5500 mass spectrometer (Sciex) operated with negative electrospray ionization (ESI) in multiple reaction monitoring (MRM) mode. Detailed instrumental parameters, analytical methods, and internal standard spiking procedures are described in Sections S3 and S4 of the Supporting Information. The 24 PFAS mixture was measured using an online SPE method on an Agilent 6460 triple quadrupole liquid chromatography–tandem mass spectrometer.^43,44^ Linear and branched isomers were measured separately, and the reported partition coefficients represent the combined sum of these isomers in the mixture.

### 2.7 Statistical Analysis

Statistical analyses were performed using Prism 10.2.1 (GraphPad, San Diego, CA). A two-way ANOVA followed by Tukey’s test for multiple comparisons was used for all data. For temporal blood concentration analysis, a linear mixed-effects model was applied using restricted maximum likelihood estimation to account for repeated measurements within individual animals. The model included dose and genotype as fixed effects and individual animal variability as a random effect, with the Geisser–Greenhouse correction applied to account for deviations from sphericity. Tukey’s post hoc test was used for multiple comparisons. This two-way ANOVA approach was also applied to the toxicokinetic parameters derived from the blood concentration–time profiles and tissue distribution patterns to account for both within replicate and between-genotype effects.

### 2.8 Toxicokinetic Analysis

The blood concentrations of PFOS measured over time for the 5 mg/kg, 0.5 mg/kg, and 0.1 mg/kg treatment groups were analyzed to determine key toxicokinetic parameters and compare these between FABP knockouts and wild-type mice. Because absorption and distribution was rapid, the blood concentrations were ln-transformed to fit elimination rate constants (*k*) using the first-order elimination equation *C*(*t*) = *C*_max_·e^–*k·(t-*T_max_)^, where *C*(*t*) is the blood concentration at time *t*,*C*_max_ is the maximum blood concentration, and T_max_ is the time to reach *C*_max_. Corresponding elimination half-lives (*t*_1/2_) were subsequently calculated as *t*_1/2_ = ln(2)/*k*. *C*_max_ and T_max_ were directly obtained from the blood concentration data set. The apparent volume of distribution (V_d_/F) that accounts for the bioavailability (F) of PFOS was calculated as V_d_/F = *D*/(*C*_max_), where *D* is the administered oral dose (mg/kg). The apparent clearance (CL/F) was determined as CL/F = *k*·V_d_/F. The total exposure of the test animals to PFOS over 65 days was calculated as area under the curve (AUC) using the trapezoidal method applied to the blood concentration–time data.

## 3 Results and Discussion

Single PFOS doses of 0.1, 0.5, and 5 mg/kg did not affect body weight, absolute liver weight, or normalized liver weight in FABP knockout compared to their respective wild-type mice (Figure S3).

### 3.1 Temporal Blood Concentrations

The concentration–time profiles showed a rapid initial increase in blood concentrations (*C*_blood_), reaching *C*_max_ within the first one to 10 days, with the exception of I-FABP^–/–^ mice at 0.1 mg/kg, which reached *C*_max_ at day 18. followed by a gradual decline over the experimental period across all treatment groups and dosages, consistent with first-order elimination (Figure 1). Temporal *C*_blood_ did not differ significantly across FABP knockout and wild-type groups for all dosages (one-way ANOVA + Tukey’s, *p* > 0.05), except for L-FABP^–/–^ at 0.5 mg/kg PFOS (Figure 1B). However, as *C*_blood_ eventually stabilized to match levels in wild-type and I-FABP^–/–^ mice, and since this pattern was not observed at the 5 mg/kg and 0.1 mg/kg doses, the difference could be random variation.

**Figure 1:**
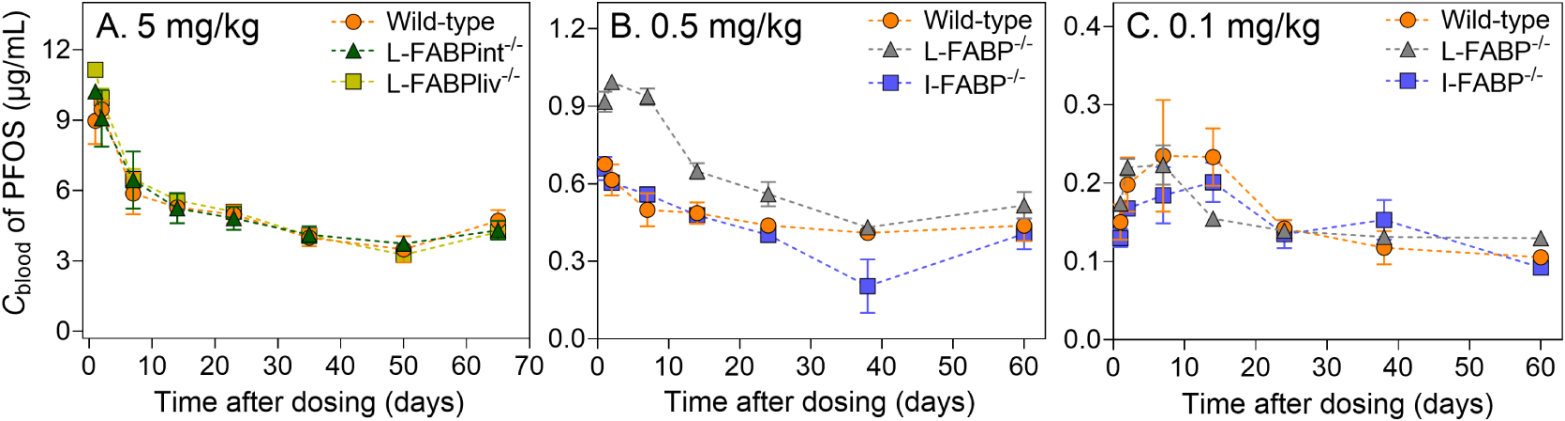
Blood concentrations measured over time in wild-type mice (L-FABP^fl/fl^ control in A; WT control in B and C.) and mice with liver-specific (L-FABPint^–/–^, L-FABPliv^–/–^) or global FABP deletion (L-FABP^–/–^, I-FABP^–/–^), after single oral administration of A. Five mg/kg, B. 0.5 mg/kg, and C. 0.1 mg/kg PFOS (*n* = 3–5).

### 3.2 Blood Toxicokinetics

The toxicokinetic parameters derived from the *C*_blood_-time profiles are reported in Table 1. For each individual dose (0.1, 0.5, 5 mg/kg), the TK parameters were consistent across the tested genotypes, showing no significant differences. The only exception was L-FABP^–/–^ that showed statistically higher *C*_max_ and thus lower V_d_ at the 0.5 mg PFOS/kg dose (Figure 1B). However, this trend was not observed for the other two doses, suggesting a random observation.

**Table 1:** Toxicokinetic Parameters Derived from the Blood Concentration–Time Profiles for Different Genotypes at Three PFOS Doses^a^

		5 mg/kg			0.5 mg/kg			0.1 mg/kg		
Parameters	Unit	Wild-type	L-FABP liv^–/–^	L-FABP int^–/–^	Wild-type	L-FABP^–/–^	I-FABP^–/–^	Wild-type	L-FABP^–/–^	I-FABP^–/–^
*t*_1/2_	day	36 ± 8^a^	35 ± 5^a^	43 ± 6^a^	47 ± 11^a^	50 ± 13^a^	49 ± 16^a^	75 ± 44^a^	72 ± 22^a^	84 ± 31^a^
T_max_	day	1.7 ± 0.6^a^	1.3 ± 0.6^a^	1.3 ± 0.6^a^	1.3 ± 0.6^a^	3.7 ± 2.9^a^	3.3 ± 2.5^a^	6 ± 5.9^a^	4 ± 2.7^a^	18 ± 13.2^a^
*C*_max_	μg/mL	9.6 ± 0.9^a^	10.9 ± 0.4^a^	10.4 ± 0.5^a^	0.69 ± 0.06^a^	0.95 ± 0.07^b^	0.67 ± 0.08^a^	0.25 ± 0.09^a^	0.24 ± 0.04^a^	0.23 ± 0.07^a^
AUC	μg/mL × day	544 ± 156^a^	523 ± 44^a^	564 ± 24^a^	52 ± 4 ^ab^	72 ± 13^a^	38 ± 14^b^	20 ± 7^a^	21 ± 5^a^	19 ± 6^a^
V_d_/F	mL/kg	571 ± 112^a^	457 ± 15^a^	489 ± 10^a^	740 ± 22^a^	545 ± 44^b^	769 ± 107 ^a^	662 ± 163^a^	560 ± 49^a^	802 ± 196^a^
CL/F	mL/kg/day	11.1 ± 2.7^a^	9.2 ± 1.5^a^	8 ± 0.9^a^	11.3 ± 2.9^a^	7.9 ± 1.9^a^	11.4 ± 2.2^a^	8.8 ± 6.5^a^	5.7 ± 1.5^a^	7.6 ± 3.7^a^

a All values are means ± standard deviations for *n* = 3–5 animals. Statistical comparisons between genotypes within the same dose were made using two-way ANOVA with Tukey’s post hoc test. Values sharing the same letter are not significantly different from each other (*p* ≥ 0.05).

Although not statistically significant, elimination half-lives (*t*_1/2_) tended to be dose-dependent, being shorter at the higher dose (35–43 days for 5 mg/kg) and progressively increasing with decreasing doses, from 47 to 50 days at 0.5 mg/kg to 72–84 days at 0.1 mg/kg. Similarly, *C*_max_ was reached more quickly at higher doses, with T_max_ extending at lower doses, while the differences in *C*_max_ and AUC among the doses aligned with the differences in administered doses. Interestingly, V_d_/F tended to increase as the dose decreased, and consequently, apparent clearance (CL/F) was higher at the higher doses, suggesting dose-dependent kinetics. Measured *t*_1/2_ for the 5 mg/kg dose were comparable to *t*_1/2_ measured previously, whereas V_d_/F and CL/F values were ∼2 times higher in our study as compared to a previous study that applied a single oral dose of 1 mg PFOS/kg.^11^

Our data on temporal *C*_blood_ of PFOS and the derived toxicokinetic parameters suggest that L-FABP or I-FABP deletion does not consistently alter systemic PFOS exposure or volume of distribution across doses. However, *C*_max_ and V_d_/F were significantly different in the L-FABP^–/–^ group at 0.5 mg/kg compared to WT and I-FABP^–/–^, whereas no other dose or genotype combination showed significant toxicokinetic differences. Notably, serum albumin levels were modestly lower in L-FABP^–/–^ mice (∼19%, Figure S6B), but this would be expected to increase V_d_/F and reduce *C*_max_, contrary to our findings. While the higher *C*_max_ and lower V_d_/F observed in L-FABP^–/–^ mice at 0.5 mg/kg were statistically significant, the absence of similar differences at 0.1 and 5 mg/kg, combined with consistent half-lives and tissue distribution, suggests this is more likely due to random variation than a biologically relevant effect. The differences in toxicokinetics across doses, though not statistically significant, indicate that other factors are likely to play a more important role in the systemic circulation and elimination of PFOS. To analyze the effects of FABP binding to PFOS distribution out of the systemic circulation, PFOS levels in various tissues were measured at the end of the experiments.

### 3.3 Tissue Distribution Patterns

The tissue distribution patterns of PFOS were largely unaffected by the deletion of FABP in knockout mice compared to their respective wild-type controls across all dose levels (Figure 2). Although PFOS concentrations were generally highest in the global L-FABP knockout group at 0.5 and 0.1 mg/kg, these differences were not statistically significant (Figure 2B,C). At the highest dose (5 mg/kg), only one statistically significant difference was observed—liver PFOS levels in L-FABPint^–/–^ mice were elevated compared to L-FABPliv^–/–^ mice (Figure 2A). When tissue concentration ratios were derived (Figure S4), these trends disappeared, suggesting that the observed variations may be due to differences in dosing or body weight rather than a genotype effect. Overall, there were no significant differences in tissue concentration ratios between genotypes, indicating that FABP is not critical in mediating the distribution of PFOS to the tissues tested herein.

**Figure 2:**
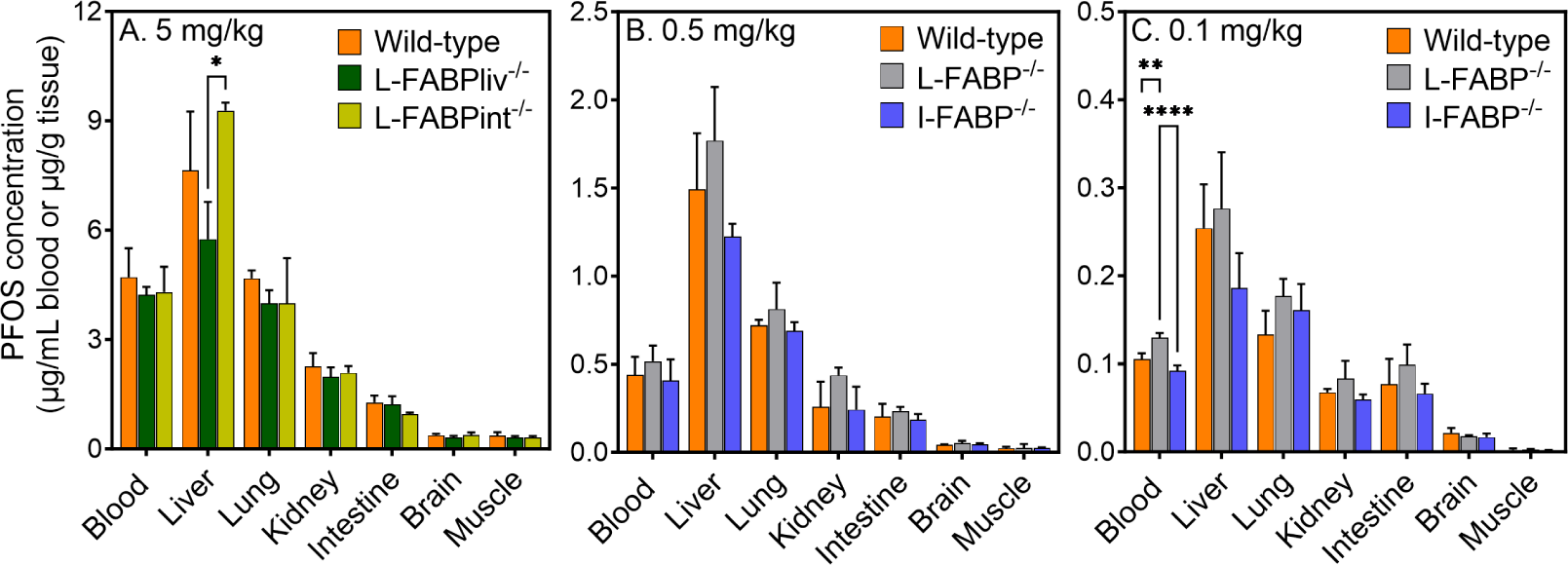
PFOS tissue concentrations measured in tissues of wild-type mice and mice with local (L-FABPint^–/–^, L-FABPliv^–/–^) or global FABP deletion (L-FABP^–/–^, I-FABP^–/–^), 60–65 days after a single oral administration of A. 5 mg/kg, B. 0.5 mg/kg, and C. 0.1 mg/kg PFOS (*n* = 3–5). L-FABP^fl/fl^ control in A; WT control in B and C. Statistical comparisons were made using two-way ANOVA with Tukey’s post hoc test (*p < 0.05, **p < 0.01, ***p < 0.001, ****p < 0.0001).

In control mice, the liver/blood concentration ratio at 0.5 mg/kg was significantly higher than at the 5 and 1 mg/kg doses (Figure S5A). Additionally, kidney/blood, brain/blood, and muscle/blood ratios were significantly higher at 0.1 mg/kg compared to 5 mg/kg doses. Notably, such differences were not observed in mice with global L-FABP and I-FABP deletion (Figure S5B,C). These findings again indicate dose-dependent PFOS toxicokinetics, which aligns with the trend of slower elimination observed at lower doses (Figure 1, Table 1). This observation aligns with recent findings of nonmonotonic dose–response relationships observed in *in vitro* hepatocyte studies^45^ and among firefighters with occupational PFAS exposure.^46^ Our data suggest that dose-dependent toxicokinetics may contribute to these responses, emphasizing the need for mechanistic studies to investigate the saturation of protein binding and the roles of uptake and efflux transporters across PFAS exposure levels, ranging from baseline environmental exposures to occupational and high-dose conditions in animal studies.

Across all genotypes and doses, the liver consistently accumulated the highest PFOS levels, followed by the lung, blood, kidney, intestine, brain, and muscle tissue. These observations confirm liver tissue as the primary site of PFOS accumulation.^13,14,41,47^ It is also interesting to note the relatively high distribution of PFOS to lung tissue, even though the chemical was administered orally. This observation aligns with relatively high PFOS levels measured in human lung tissue.^14^ The fact that PFOS reaches the lungs despite the lack of inhalation exposure suggests that PFOS may have a high affinity for lung tissue, which was recently suggested in binding studies.^41^

### 3.4 Excretion through Urine and Feces

PFOS excretion through urine and feces was generally comparable between wild-type and across FABP knockouts (Figure 3). This suggests that the deletion of FABP does not significantly alter the disposition of PFOS into these excretory pathways, aligning with the observed similar circulatory toxicokinetics and tissue distribution patterns observed across the genotypes. Notably, PFOS levels in feces were 4–5 times higher than in urine, suggesting biliary excretion as the primary elimination route following oral administration in mice. This contrasts with a previous mouse study,^11^ where urinary excretion was the dominant elimination pathway, but aligns with the fecal/urinary elimination ratios of ∼5 and 3.65 observed for PFOS in rats^48^ and humans.^49^ There was a slight trend of decreasing PFOS levels in both urine and feces over time, consistent with the temporal decline in blood concentrations. L-FABP^–/–^ mice exhibited slightly higher PFOS levels in urine and feces at the 0.5 mg/kg dose after 16 days, which correlates with the higher *C*_blood_ observed at earlier time points (Figure 1B). However, these differences were not statistically significant, reinforcing the conclusion that FABP deletion has no significant impact on PFOS excretion, suggesting that other toxicokinetic pathways are more influential in determining PFOS elimination. PFOS levels in urine and feces were also measured in the 0.1 mg/kg dose group but were consistently below detection limits across all genotypes and time points; thus, these data are not shown.

**Figure 3:**
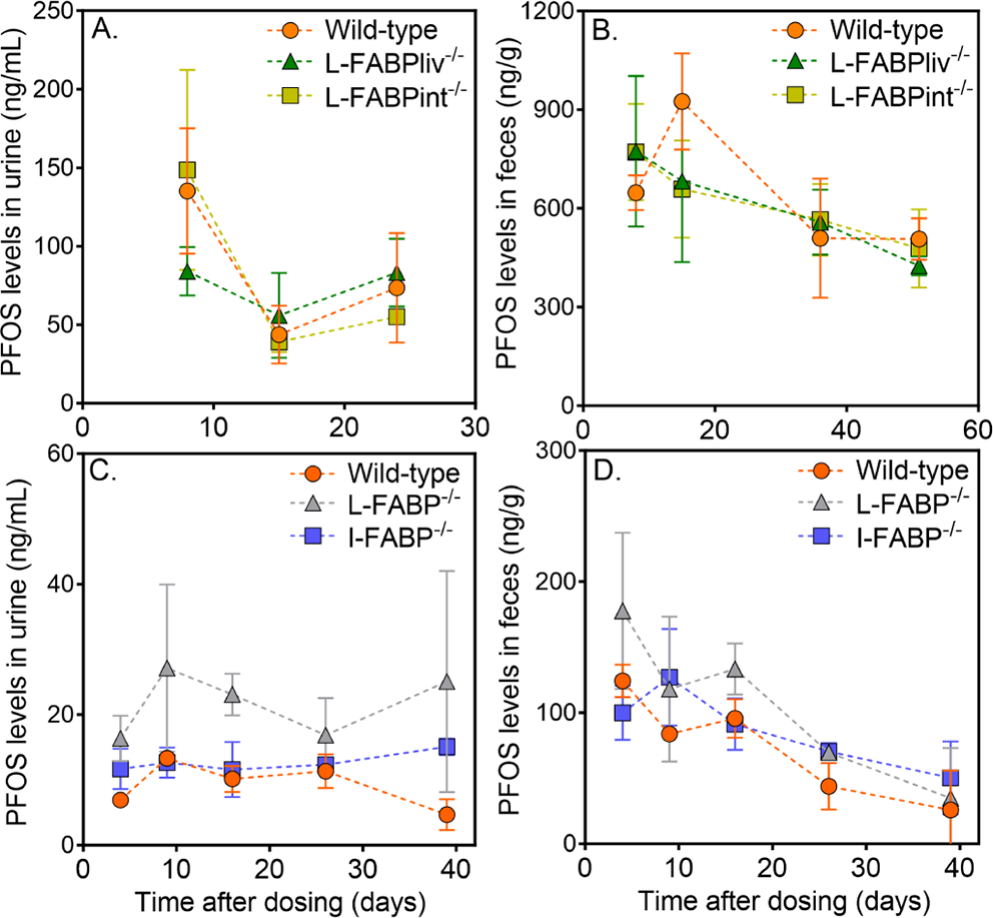
PFOS concentrations in urine and feces over time in wild-type mice and mice with local (L-FABPint^–/–^, L-FABPliv^–/–^) or global FABP deletion (L-FABP^–/–^, I-FABP^–/–^). Mice were dosed with 5 mg/kg (A, B) or 0.5 mg/kg (C, D) of PFOS (*n* = 3–5).

### 3.5 PFAS Binding to Liver Tissues

Figure 4 shows the fold-difference in liver partition coefficients (*K*_liver_) measured for liver tissues sampled from wild-type mice and global L-FABP knockout (L-FABP^–/–^) mice. These *K*_liver_ values were obtained using C18 fiber solid-phase microextraction (SPME) with liver tissue homogenates from PFOS-free wild-type and global L-FABP knockout mice. For 15 of the 19 PFAS tested, the difference in *K*_liver_ between wild-type and knockout mice was less than 20%, indicating no considerable difference in partitioning. Notably, 14 PFAS were above the 1:1 line, indicating slightly higher binding to FABP-containing liver tissues. However, these small differences might not translate into significant *in vivo* effects, as was the case for PFOS (Figures 2 and S2). The four PFAS that showed more than a 20% difference in *K*_liver_ between genotypes all contain sulfonate or sulfonamide headgroups. These included PFDS (+32%), PFNS (+55%), 10:2 FTS (+26%), and FOSA (−21%). These findings suggest that FABP may play a role in the liver accumulation of these specific chemicals, suggesting testing these PFAS in future experiments with FABP knockouts.

**Figure 4:**
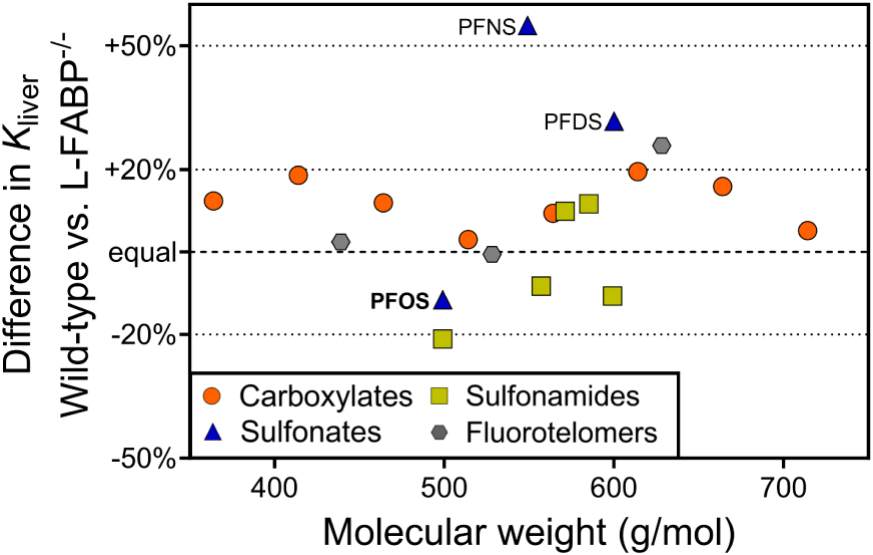
Percentage difference in liver partition coefficients measured for liver tissues sampled from wild-type mice compared to mice with global L-FABP deletion (L-FABP^–/–^). PFAS classes are indicated by the color and shape of symbols, and sorted by their molecular weight.

While it is possible that other FA-binding proteins may be compensating for the absence of the L-FABP in liver and/or intestine and for I-FABP in intestine, it is noteworthy that in the global and conditional FABP knockouts, no upregulation of other FABPs was observed (Figure S1).^30–32^ Interestingly, in both the intestine-specific and liver-specific knockouts, serum albumin levels were modestly elevated (∼40–44% higher than wild-type, Figure S6A), while global knockout of L-FABP was associated with an ∼19% reduction in serum albumin (Figure S6B). Despite these changes, because albumin is secreted and extracellular, and spatially segregated from the cytosolic FABPs, such shifts are unlikely to represent true intracellular compensation or to substantially influence intracellular PFAS partitioning.

### 3.6 Implications for PFAS Toxicokinetics in Humans

This study is the first to examine circulatory exposures and tissue distribution of PFOS after oral administration in mice lacking L-FABP or I-FABP. Despite *in vitro* and binding studies suggesting FABP as an important binding protein for PFAS,^18,24–28^ the lack of significant differences in PFOS distribution and elimination between FABP knockouts and wild-type mice indicates that FABP is not a crucial determinant of PFOS toxicokinetics. This suggests that other mechanisms, such as binding to phospholipids^50^ and/or structural proteins^51^ may be responsible for the high concentrations of PFOS observed in the liver. Additionally, permeability and transporter-mediated uptake are suggested key mechanisms for PFAS tissue distribution and renal and biliary excretion.^52–57^ The role of membrane transporters such as organic anion transporters (OAT) and organic anion transporting polypeptides (OATP) could be evaluated in future knockout experiments using the framework from our study.

The herein reported blood exposure and tissue distribution data can be used to evaluate the performance of physiologically based toxicokinetic (PBTK) models developed to analyze the mechanisms of PFAS toxicokinetics *in vivo*. Given that PBTK models have been developed for both mice and rats, it would be valuable to compare our data to model simulations in presence and absence of FABP binding in liver and other tissues.

This study provides important insights into the relative importance of FABP in toxicokinetics of PFOS and PFAS as a chemical class. When extrapolating our findings to human exposures, the fact that FABP levels in human livers are approximately twice as high as in mice should be considered.^58^ Additionally, species differences in the binding affinity of PFAS to FABPs have been observed, with PFAS binding more strongly to human FABP than to rodent FABP.^59^ Both the abundance of FABP in various organs as well as their capacity to bind PFAS should be considering when extrapolating ours study findings to human exposures. However, FABP is less abundant in the human liver compared to other binding molecules like phospholipids and structural proteins,^60^ which may explain its limited impact on tissue distribution *in vivo*.

While our data suggest that FABP does not play a significant role in PFAS toxicokinetics, it does not examine whether or how the absence of FABP would affect toxicological outcomes associated with PFAS exposures, such as hepatic lipid accumulation or oxidative stress. Toxicological and histopathological changes in FABP knockout versus wild-type mice have been analyzed and will be published in a separate manuscript. Additionally, a limitation of the study herein is that relatively high concentrations of single PFAS were used, whereas real-world human exposures typically involve lower concentrations, repeated exposures, and complex PFAS mixtures. Nonmonotonic dose–response relationships have been observed in both *in vitro* and epidemiological studies,^45,46^ suggesting nonlinearity of tissue binding and/or transporter-mediated distribution. This underscores the need for future studies to investigate PFAS toxicokinetics at environmentally relevant concentrations within complex mixtures, recreating chronic human exposures with a repeated dosing regimen, and additional PFAS. Such studies will help determine whether similar dose-dependent patterns persist and further clarify the role of FABP in the context of human exposures. Additionally, comorbidities like metabolic dysfunction-associated steatotic liver disease (MASLD)^61^ and obesity^62^ have been associated with altered FABP levels in the liver, which could affect the susceptibility of affected individuals to PFAS toxicities. Future research should explore the interplay between FABP deficiency, PFAS toxicokinetics, and the role of FABP in modulating toxicological outcomes in the context of these comorbid conditions. While we included both systemic and liver-specific L-FABP knockouts at different doses, a fully systematic dose–response comparison across all exposure levels could further confirm our findings. However, given the consistent lack of toxicokinetic differences across genotypes and doses—with the exception of an isolated variation at 0.5 mg/kg—we are confident that FABPs do not play a significant role in PFOS toxicokinetics under the tested conditions.

## Supplementary Material



## Notes

Sample preparation; PFOS extraction procedure; instrument analysis and quality assurance; body and liver weights; tissue/blood concentration ratios (PDF)
