# Supplementary material for: Liver and Intestinal Fatty Acid Binding Proteins Are Not Critical for Perfluorooctanesulfonate (PFOS) Tissue Distribution and Elimination in Mice
Source: Chem Res Toxicol. 2025 Aug 19;38(9):1576–84. doi: 10.1021/acs.chemrestox.5c00199 (PMC12442221; doi:10.1021/acs.chemrestox.5c00199)
Supplement: Supplementary file 1 [file tx5c00199_si_001.pdf]

Supporting Information

Liver and intestinal fatty acid binding proteins are not critical for perfluorooctane sulfonate (PFOS) tissue distribution and elimination in mice

Seyed Mohamad Sadegh Modaresi[1], Jitka Becanova[2], Simon Vojta[2], Sangwoo Ryu[1], Emily M. Kaye[1], Juliana Agudelo[1], Anastasia Diolintzi[3], Olga Skende[1], Judith Storch[3], Fabian C. Fischer[1,4,*], Angela Slitt[1,*]

[1]Department of Biomedical and Pharmaceutical Sciences, University of Rhode Island, Kingston, Rhode Island, 02881, United States.
[2]Graduate School of Oceanography, University of Rhode Island, Kingston, Rhode Island, 02881, United States.
[3]Department of Nutritional Sciences, Rutgers University, New Brunswick, New Jersey, 08901, United States.
[4]Harvard John A. Paulson School of Engineering and Applied Sciences, Harvard University, Cambridge, Massachusetts 02138, United States.

Corresponding Authors:

1. Angela Slitt, Ph.D.
University of Rhode Island
395D, Avedisian Hall
7 Greenhouse Rd., Kingston
RI 02881, USA
Phone: 401-874-5020
Email: aslitt@uri.edu

2. Fabian C. Fischer, Ph.D.
University of Rhode Island
495Q, Avedisian Hall
7 Greenhouse Rd., Kingston
RI 02881, USA
Phone: 401-874-5594
Email: fabian.fischer@uri.edu

Content overview: 15 Pages, 7 Figures, 5 Tables

Section	Contents	Page
S-1.	Liver Fatty Acid Binding Protein 1-5 content in liver	S-3
S-2.	Sample Preparation	S-3
S-3.	PFOS extraction procedure	S-4
S-4.	Instrument Analysis and Quality Assurance	S-5
S-5.	Body and liver weights	S-7
S-6.	Tissue/blood concentration ratios	S-8
S-7.	Serum albumin concentrations	S-9
S-8.	Statistical comparisons	S-10
S-9.	References used in Supporting Information	S-15

S-1. Liver Fatty Acid Binding Protein 1-5 content in liver.

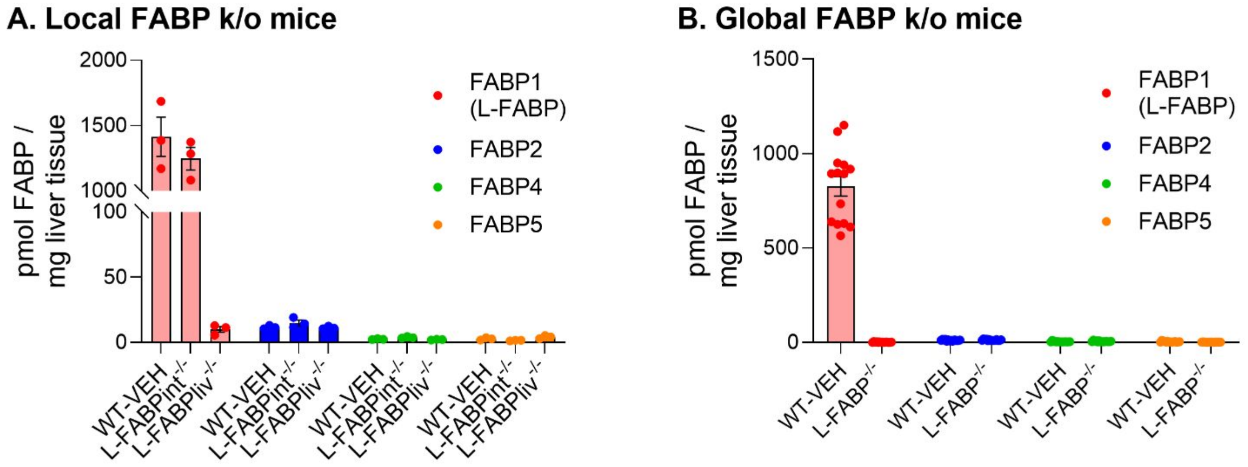

Figure S1. Quantification of FABP1–5 in mouse liver tissue using targeted proteomics. (A) Liver protein concentrations of FABP1 (L-FABP), FABP2, FABP4, and FABP5 in wild-type mice (WT-VEH), intestine-specific L-FABP knockout (L-FABPint$^{-/-}$), and liver-specific L-FABP knockout (L-FABPliv$^{-/-}$) mice. (B) Liver FABP concentrations in wild-type mice and global L-FABP knockout (L-FABP$^{-/-}$) mice. FABP1 was the dominant isoform detected in wild-type livers and was specifically depleted in liver- or globally-deficient L-FABP knockout mice, confirming genotype-specific ablation. FABP2, FABP4, and FABP5 remained at low levels and were unaffected by L-FABP deletion.

S-2. Sample Preparation.

For tracing PFOS blood elimination kinetics, small volumes of blood were obtained from animals by through cheek pouch submandibular vein bleeding with a 5 mm lancet (Medipoint, Utrecht, Utrecht, The Netherlands). Large volumes of blood were collected by cardiac puncture using 1 ml plastic syringes equipped with 25-gauge needles prior to euthanasia. Predetermined collection time points over the course of the study are shown in the caption of the figures. To collect urine and feces samples, animals were put in mice specific metabolic cages with mesh bottoms for about 4 h making the samples free from contamination. Before each timepoint, metabolic cages were rinsed with soap and tap water, and then airdried. The urine samples were collected directly to the 1.5 ml polypropylene centrifuge tubes. Feces samples were manually collected from metabolic cages using tweezers and stored in 1.5 ml polypropylene centrifuge tubes. Tissues were also harvested and snap frozen in liquid nitrogen during necropsy and were stored at -80 °C until analysis.

S-3. PFOS extraction procedure.

The extraction was performed using Phenomenx roQ QuEChERS kits following the manufacturer's instructions with slight modifications[1]. In brief, ~20 mg of tissue was homogenized in 2 ml Omni Hard Tissue Homogenizing tubes containing 1.4 mm ceramic beads, and 400 µl deionized water and acetonitrile (1:1 ratio) spiked with a fixed amount of stable isotope-labeled internal standard. Using an Omni Bead Ruptor Elite (Omni International, Kennesaw, GA), the mixture was homogenized for 30 s at 4 m/sec. Next, the homogenate was transferred to centrifuge tube containing ~100 mg salt from extraction packet kit (4 g MgSO4 + 1 g NaCl), vortexed for 30 S and centrifuged (10 minutes, 4000 × g) at room temperature to make a distinct separation between acetonitrile, water, and solid layer. In the next step, ~200 µl of acetonitrile was transferred to centrifuge tube containing ~30 mg of dSPE kit for further sample clean-up. The sample was vortexed for 30 S before centrifugation (10 minutes, 4000 × g). Finally, 50 µl of the acetonitrile was reconstituted with 50 µl of deionized water and vortexed for 30 s prior to LC–MS/MS analysis. Whole blood was used for the extraction of PFOS according to the method described by Usui et al. (2012) with slight modifications[2]. In brief, three-fold diluted blood with water was mixed with the internal standard solution was added to the QuEChERS salt packet extraction kit (containing magnesium sulfate and sodium chloride), vortexed, and partitioned into three layers by centrifugation. In the next step, the top layer (acetonitrile) was transferred into a centrifuge tube containing roQ QuEChERS dSPE (primary secondary amine, and magnesium sulfate) and mixed for purification. After the centrifugation, supernatant was injected into LC–MS/MS. The same steps were followed for preparing urine samples while fecal samples were first homogenized, and then the abovementioned steps were followed, and the final supernatant was run for PFOS analysis.

PFOS recoveries from tissues using the extraction method were determined by spiking the tissues with 13C-labeled PFOS prior to extraction according to the protocol and comparing the extracted 13C-PFOS to the matrix-spiked standard. The extraction method performance was verified with recoveries of PFOS within ~ ± 40% (Table S1).

Table S1. Recoveries (%) of 13C-PFOS from various tissues and excreta (mean ± SD). Values are expressed as percentages to reflect the efficiency of the applied extraction protocol. Blood n = 68, liver, intestine, lung, kidney, brain, muscle n = 10, urine n = 42, feces n = 33.

Tissue/excreta	13C-PFOS recovery (%)
Blood	113% ± 13%
Liver	79% ± 3%
Intestines	64% ± 6%
Lung	106% ± 7%
Kidney	119% ± 3%
Brain	116% ± 4%
Muscle	116% ± 11%
Urine	132% ± 52%
Feces	123% ± 10%

S-4. Instrument Analysis and Quality Assurance.

A LC-MS/MS system with a CTC PAL autosampler (LEAP Technologies,Carrboro, NC), a 1290 binary pump (Agilent, Santa Clara, CA) with Kinetex, 2.6 um C18 30 x 2.1mm (Phenomenex, Torrance, CA), and a triple quadrupole 5500 mass spectrometer (Sciex, Foster City, CA) was used for sample analysis. Samples and standards were injected (10 µL) at 40 °C and a linear UPLC gradient was performed from 90% mobile phase A (0.1% formic acid in water) to 95% mobile phase B (0.1% formic acid in acetonitrile) over 1.2 min at a flow rate of 0.5 mL/min to elute the compound. Multiple reaction monitoring was used to detect ion transitions of analytes. The MRM ion pairs used for PFOS quantification were 499.1/80.1 and for the PFOS standard was 502.9/79.8 (parent ion m/z / fragment ion m/z) in conjunction with a matrix matched calibration curve and an isotope dilution method with mass labeled analogs used as surrogate standards in both, the calibration curve and analyzed samples to determine unknown concentration. Analyst version 1.6.2 and MultiQuant version 3.0.2 (Applied Biosystems, Foster City, CA) were applied for data acquisition and quantitation. All calculations were based on area ratios (analyte peak area/IS peak area). In this study, the Limit of Detection (LOD) and Limit of Quantification (LOQ) were calculated based on the standard deviation of the residuals (σ) and the slope (S) of the calibration curve derived from a linear regression of concentration versus analyte peak area. The calculated LOD of PFOS was 1.30 ng/ml and the calculated LOQ was 3.90 ng/mL.

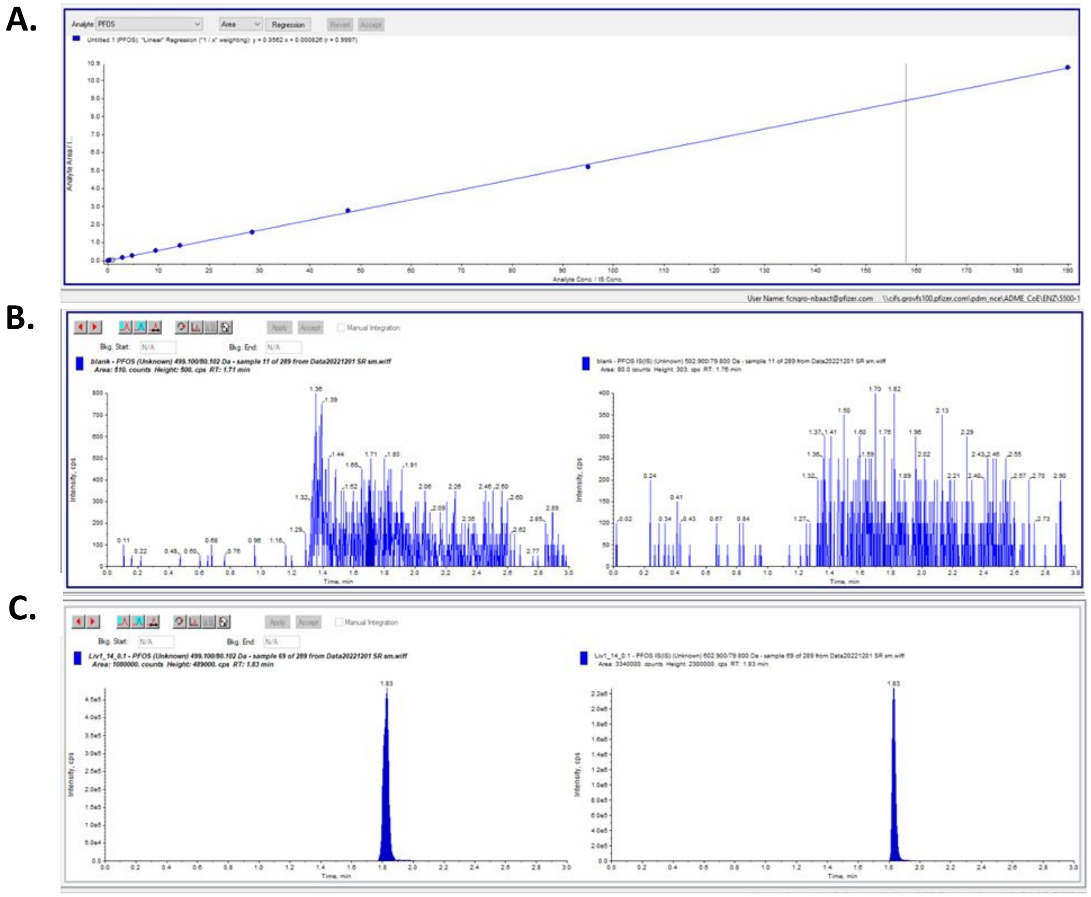

Figure S2. LC-MS/MS analysis of PFOS. Example of a standard curve of LC-MS/MS measurement of PFOS (A). Example of LC-MS/MS chromatograms of the blank (B). Example of LC-MS/MS chromatograms of the PFOS extracted from the liver of the mice exposed to a single dose of 0.1 mg PFOS/kg body weight at day 14 (C).

S-5. Body and liver weights.

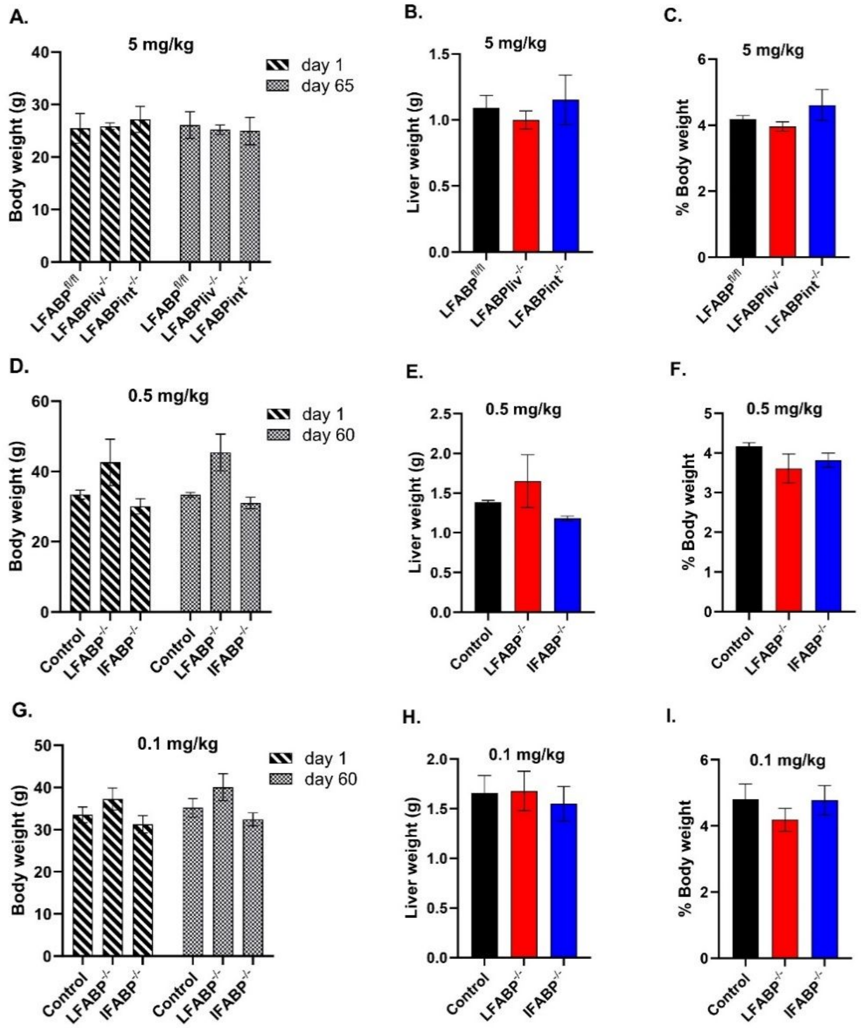

Figure S3. Effect of PFOS exposure on body weight, and liver weight. Body weight, liver weight, and the liver/body weight ratio of mice after treatment with a single oral dose of 5 (A, B, C), 0.5 (D, E, F), and 0.1 mg PFOS /kg body weight (G, H, I). No significant difference between knock out mice and control were observed. The error bars represented as ±SE. N = 3-5.

S-6. Tissue/blood concentration ratios.

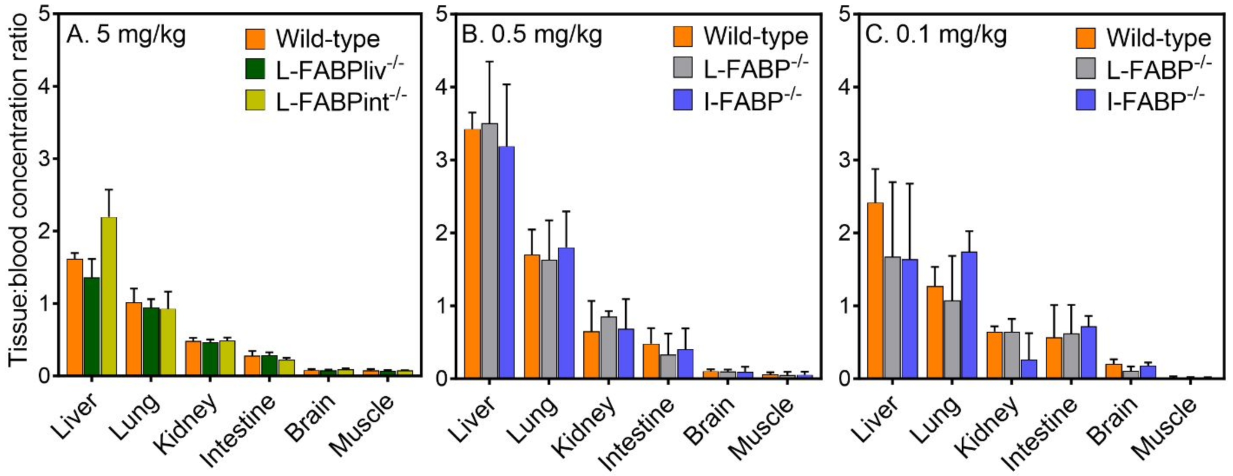

Figure S4. PFOS tissue:blood concentration ratios measured in wild-type mice and mice with local (L-FABPint$^{-/-}$, L-FABPliv$^{-/-}$) or global FABP deletion (L-FABP$^{-/-}$, I-FABP$^{-/-}$), 60-65 days after a single oral administration of A. 5 mg/kg, B. 0.5 mg/kg, and C. 0.1 mg/kg PFOS (*n*=3-5). L-FABP$^{fl/fl}$ control in A; WT control in B and C. Statistical comparisons were made using two-way ANOVA with Tukey's post-hoc test (*p < 0.05, **p < 0.01, ***p < 0.001, ****p < 0.0001).

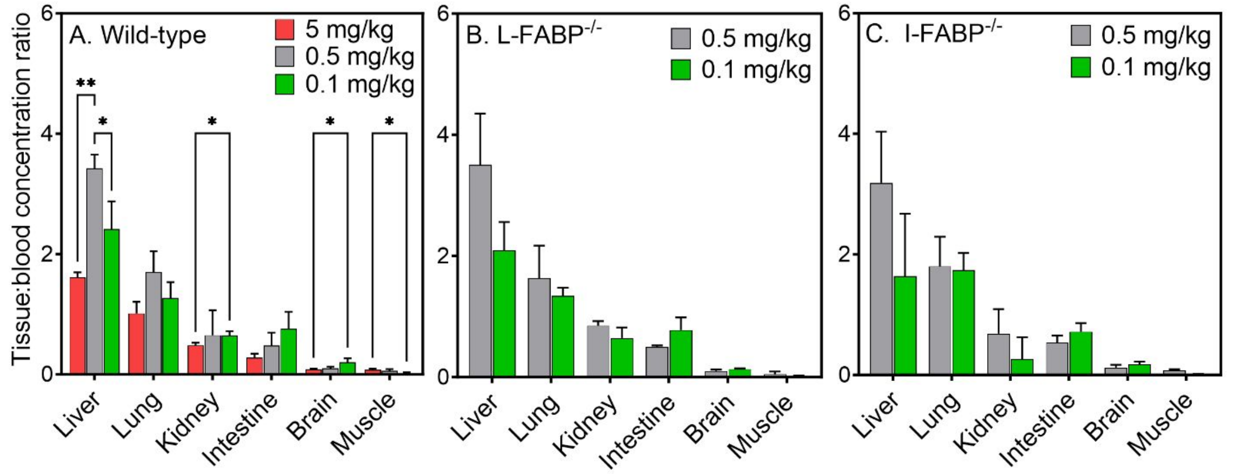

Figure S5. PFOS tissue:blood concentration ratios measured in A. wild-type (FABP$^{+/}$) mice and mice with global B. L-FABP (L-FABP$^{-/-}$) and C. I-FABP deletion (I-FABP$^{-/-}$), 60-65 days after a single oral administration of A. 5 mg/kg, B. 0.5 mg/kg, and C. 0.1 mg/kg PFOS (*n*=3-5). L-FABP$^{fl/fl}$ control in A; WT control in B and C. Statistical comparisons were made using two-way ANOVA with Tukey's post-hoc test (*p < 0.05, **p < 0.01, ***p < 0.001, ****p < 0.0001).

S-7. Serum albumin concentrations.

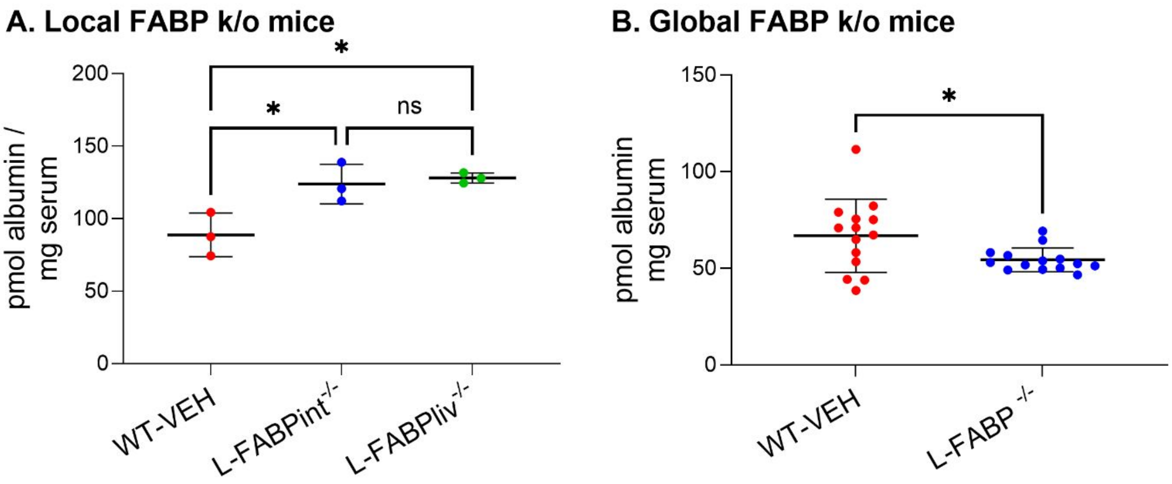

Figure S6. Quantitative proteomics of serum albumin in local and global L-FABP knockout mice. (A) Serum albumin concentrations (pmol/mg serum) measured by targeted proteomics in wild-type (WT-VEH), intestine-specific L-FABP knockout (L-FABPint$^{-/-}$), and liver-specific L-FABP knockout (L-FABPliv$^{-/-}$) mice. Albumin levels were significantly elevated in both knockout models compared to WT, with L-FABPint$^{-/-}$ and L-FABPliv$^{-/-}$ mice showing 40% and 44% higher levels, respectively. No significant difference was observed between the two knockout groups. Differences were assessed by one-way ANOVA followed by Tukey's HSD test. (B) Global L-FABP knockout (L-FABP$^{-/-}$) mice exhibited significantly reduced serum albumin levels compared to WT, with an average decrease of 19%. Group comparison was performed using Welch's t-test due to unequal variances. *$p < 0.05$; ns = not significant. *$p < 0.05$; ns = not significant.

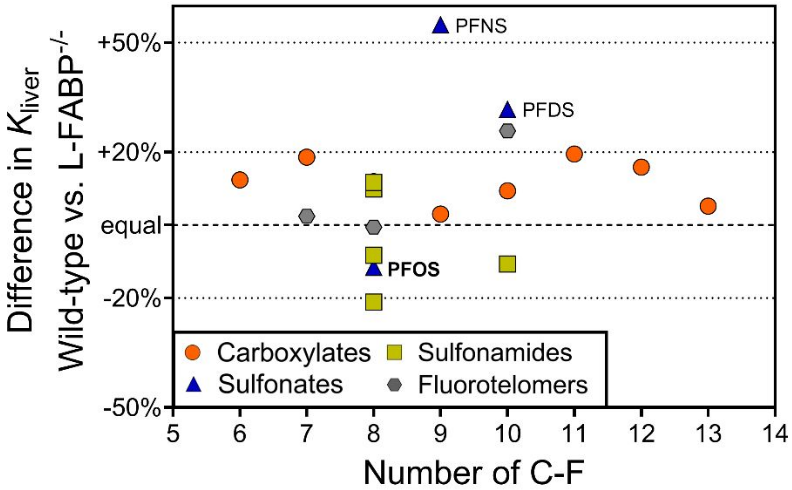

Figure S7. Percentage difference in liver partition coefficients measured for liver tissues sampled from wild-type mice compared to mice with global L-FABP deletion (L-FABP$^{-/-}$). PFAS classes are indicated by the color and shape of symbols, and sorted by their fluorinated carbon chain length.

S-8. Statistical comparisons.

Table S2. Statistical comparisons of toxicokinetic parameters between genotypes at 5 mg/kg PFOS. Comparisons were performed using two-way ANOVA with Tukey's post-hoc test. Reported values include mean differences, 95% confidence intervals, adjusted p-values, and significance (ns = not significant).

Parameter	Comparison	Mean Diff.	95% CI of Diff.	Adjusted P Value	Significance
Half-life (d)	Control vs. L-FABPliv$^{-/-}$	1.527	-19.32 to 22.37	0.9558	ns
Half-life (d)	Control vs. L-FABPint$^{-/-}$	-6.653	-27.63 to 14.32	0.5335	ns
Half-life (d)	L-FABPliv$^{-/-}$ vs. L-FABPint$^{-/-}$	-8.180	-24.41 to 8.053	0.2734	ns
C_{max} (µg/mL)	Control vs. L-FABPliv$^{-/-}$	-1.337	-3.994 to 1.321	0.2118	ns
C_{max} (µg/mL)	Control vs. L-FABPint$^{-/-}$	-0.783	-3.275 to 1.708	0.4857	ns
C_{max} (µg/mL)	L-FABPliv$^{-/-}$ vs. L-FABPint$^{-/-}$	0.553	-0.762 to 1.869	0.3630	ns
T_{max} (d)	Control vs. L-FABPliv$^{-/-}$	0.333	-1.347 to 2.013	0.7727	ns
T_{max} (d)	Control vs. L-FABPint$^{-/-}$	0.333	-1.347 to 2.013	0.7727	ns
T_{max} (d)	L-FABPliv$^{-/-}$ vs. L-FABPint$^{-/-}$	0.000	-1.680 to 1.680	>0.9999	ns
V_d/F (mL/kg)	Control vs. L-FABPliv$^{-/-}$	114.2	-256.4 to 484.7	0.3689	ns
V_d/F (mL/kg)	Control vs. L-FABPint$^{-/-}$	82.0	-294.1 to 458.0	0.5323	ns
V_d/F (mL/kg)	L-FABPliv$^{-/-}$ vs. L-FABPint$^{-/-}$	-32.2	-72.4 to 8.017	0.0923	ns
CL/F (mL/kg/d)	Control vs. L-FABPliv$^{-/-}$	1.903	-5.470 to 9.277	0.5991	ns
CL/F (mL/kg/d)	Control vs. L-FABPint$^{-/-}$	3.170	-5.000 to 11.34	0.3088	ns
CL/F (mL/kg/d)	L-FABPliv$^{-/-}$ vs. L-FABPint$^{-/-}$	1.267	-2.809 to 5.342	0.5073	ns
AUC (µg·d/mL)	Control vs. L-FABPliv$^{-/-}$	21.59	-455.5 to 498.7	0.9713	ns
AUC (µg·d/mL)	Control vs. L-FABPint$^{-/-}$	-19.24	-529.9 to 491.5	0.9759	ns
AUC (µg·d/mL)	L-FABPliv$^{-/-}$ vs. L-FABPint$^{-/-}$	-40.83	-158.0 to 76.29	0.4319	ns

Table S3. Statistical comparisons of toxicokinetic parameters between genotypes at 0.5 mg/kg PFOS. Comparisons were performed using two-way ANOVA with Tukey's post-hoc test. Reported values include mean differences, 95% confidence intervals, adjusted p-values, and significance (ns = not significant).

**Parameter**	**Comparison**	**Mean Diff.**	**95% CI of Diff.**	**Adjusted P Value**	**Significance**
Half-life (d)	Control vs. L-FABP$^{-/-}$	-2.100	-51.15 to 46.95	0.9785	ns
Half-life (d)	Control vs. L-FABP$^{-/-}$	-1.185	-46.00 to 43.63	0.9937	ns
Half-life (d)	L-FABP$^{-/-}$ vs. L-FABP$^{-/-}$	0.9150	-34.27 to 36.10	0.9960	ns
C_{max} (µg/mL)	Control vs. L-FABP$^{-/-}$	-264.2	-506.3 to -22.09	0.0423	*
C_{max} (µg/mL)	Control vs. L-FABP$^{-/-}$	23.00	-140.3 to 186.3	0.8380	ns
C_{max} (µg/mL)	L-FABP$^{-/-}$ vs. L-FABP$^{-/-}$	287.2	96.61 to 477.8	0.0109	*
T_{max} (d)	Control vs. L-FABP$^{-/-}$	-2.333	-11.59 to 6.920	0.4839	ns
T_{max} (d)	Control vs. L-FABP$^{-/-}$	-1.917	-6.914 to 3.081	0.4018	ns
T_{max} (d)	L-FABP$^{-/-}$ vs. L-FABP$^{-/-}$	0.4167	-6.981 to 7.814	0.9783	ns
V_d/F (mL/kg)	Control vs. L-FABP$^{-/-}$	195.4	73.73 to 317.2	0.0141	*
V_d/F (mL/kg)	Control vs. L-FABP$^{-/-}$	-29.11	-243.9 to 185.7	0.8621	ns
V_d/F (mL/kg)	L-FABP$^{-/-}$ vs. L-FABP$^{-/-}$	-224.6	-430.7 to -18.39	0.0379	*
CL/F (mL/kg/d)	Control vs. L-FABP$^{-/-}$	3.332	-15.34 to 22.00	0.5009	ns
CL/F (mL/kg/d)	Control vs. L-FABP$^{-/-}$	-0.1825	-18.64 to 18.27	0.9967	ns
CL/F (mL/kg/d)	L-FABP$^{-/-}$ vs. L-FABP$^{-/-}$	-3.514	-8.609 to 1.580	0.1532	ns
AUC (µg·d/mL)	Control vs. L-FABP$^{-/-}$	-19.65	-57.19 to 17.88	0.1912	ns
AUC (µg·d/mL)	Control vs. L-FABP$^{-/-}$	17.91	-9.006 to 44.82	0.1500	ns
AUC (µg·d/mL)	L-FABP$^{-/-}$ vs. L-FABP$^{-/-}$	37.56	4.070 to 71.05	0.0336	*

Table S4. Statistical comparisons of toxicokinetic parameters between genotypes at 0.1 mg/kg PFOS. Comparisons were performed using two-way ANOVA with Tukey's post-hoc test. Reported values include mean differences, 95% confidence intervals, adjusted p-values, and significance (ns = not significant).

Parameter	Comparison	Mean Diff.	95% CI of Diff.	Adjusted P Value	Significance
Half-life (d)	Control vs. L-FABP$^{-/-}$	3.513	-80.72 to 87.75	0.9885	ns
Half-life (d)	Control vs. L-FABP$^{-/-}$	-9.329	-92.74 to 74.08	0.9322	ns
Half-life (d)	L-FABP$^{-/-}$ vs. L-FABP$^{-/-}$	-12.84	-62.17 to 36.48	0.7375	ns
C_{max} (µg/mL)	Control vs. L-FABP$^{-/-}$	14.98	-162.2 to 192.2	0.951	ns
C_{max} (µg/mL)	Control vs. L-FABP$^{-/-}$	28.32	-146.0 to 202.7	0.8686	ns
C_{max} (µg/mL)	L-FABP$^{-/-}$ vs. L-FABP$^{-/-}$	13.34	-94.01 to 120.7	0.9268	ns
T_{max} (d)	Control vs. L-FABP$^{-/-}$	2.0	-9.432 to 13.43	0.8166	ns
T_{max} (d)	Control vs. L-FABP$^{-/-}$	-12.0	-32.45 to 8.453	0.2445	ns
T_{max} (d)	L-FABP$^{-/-}$ vs. L-FABP$^{-/-}$	-14.0	-34.67 to 6.673	0.1541	ns
V_d/F (mL/kg)	Control vs. L-FABP$^{-/-}$	102.1	-223.1 to 427.2	0.5178	ns
V_d/F (mL/kg)	Control vs. L-FABP$^{-/-}$	-140.4	-493.9 to 213.1	0.5053	ns
V_d/F (mL/kg)	L-FABP$^{-/-}$ vs. L-FABP$^{-/-}$	-242.5	-549.2 to 64.21	0.1032	ns
CL/F (mL/kg/d)	Control vs. L-FABP$^{-/-}$	3.098	-9.998 to 16.19	0.6535	ns
CL/F (mL/kg/d)	Control vs. L-FABP$^{-/-}$	1.258	-10.97 to 13.49	0.9369	ns
CL/F (mL/kg/d)	L-FABP$^{-/-}$ vs. L-FABP$^{-/-}$	-1.84	-7.566 to 3.886	0.5908	ns
AUC (µg·d/mL)	Control vs. L-FABP$^{-/-}$	-1.719	-14.32 to 10.88	0.9022	ns
AUC (µg·d/mL)	Control vs. L-FABP$^{-/-}$	0.365	-12.67 to 13.40	0.996	ns
AUC (µg·d/mL)	L-FABP$^{-/-}$ vs. L-FABP$^{-/-}$	2.084	-7.875 to 12.04	0.8213	ns

Table S5. Statistical comparisons of PFOS concentrations in different tissues between genotypes at 5 mg/kg PFOS. Comparisons were performed using two-way ANOVA with Tukey's post-hoc test. Reported values include mean differences, 95% confidence intervals, adjusted p-values, and significance (ns = not significant).

**Parameter**	**Comparison**	**Mean Diff.**	**95% CI of Diff.**	**Adjusted P Value**	**Significance**
Blood	Control vs. L-FABPliv$^{-/-}$	0.4825	-1.963 to 2.928	0.6311	ns
Blood	Control vs. L-FABPint$^{-/-}$	0.4082	-1.780 to 2.597	0.792	ns
Blood	L-FABPliv$^{-/-}$ vs. L-FABPint$^{-/-}$	-0.07425	-2.158 to 2.009	0.983	ns
Liver	Control vs. L-FABPliv$^{-/-}$	1.894	-2.375 to 6.163	0.3202	ns
Liver	Control vs. L-FABPint$^{-/-}$	-1.633	-6.949 to 3.683	0.3703	ns
Liver	L-FABPliv$^{-/-}$ vs. L-FABPint$^{-/-}$	-3.527	-6.793 to - 0.2614	0.0427	*
Lung	Control vs. L-FABPliv$^{-/-}$	0.6816	-0.2849 to 1.648	0.1231	ns
Lung	Control vs. L-FABPint$^{-/-}$	0.6751	-3.317 to 4.668	0.673	ns
Lung	L-FABPliv$^{-/-}$ vs. L-FABPint$^{-/-}$	-0.006447	-3.748 to 3.735	>0.9999	ns
Kidney	Control vs. L-FABPliv$^{-/-}$	0.2946	-0.6789 to 1.268	0.5541	ns
Kidney	Control vs. L-FABPint$^{-/-}$	0.1811	-0.8123 to 1.175	0.7488	ns
Kidney	L-FABPliv$^{-/-}$ vs. L-FABPint$^{-/-}$	-0.1134	-0.8238 to 0.5969	0.829	ns
Intestine	Control vs. L-FABPliv$^{-/-}$	0.06032	-0.5572 to 0.6778	0.9344	ns
Intestine	Control vs. L-FABPint$^{-/-}$	0.3257	-0.2672 to 0.9185	0.1601	ns
Intestine	L-FABPliv$^{-/-}$ vs. L-FABPint$^{-/-}$	0.2654	-0.4688 to 0.9995	0.3065	ns
Brain	Control vs. L-FABPliv$^{-/-}$	0.05397	-0.06738 to 0.1753	0.3502	ns
Brain	Control vs. L-FABPint$^{-/-}$	-0.009655	-0.1958 to 0.1765	0.9773	ns
Brain	L-FABPliv$^{-/-}$ vs. L-FABPint$^{-/-}$	-0.06363	-0.2502 to 0.1230	0.4549	ns
Muscle	Control vs. L-FABPliv$^{-/-}$	0.06199	-0.1829 to 0.3069	0.591	ns
Muscle	Control vs. L-FABPint$^{-/-}$	0.05675	-0.1919 to 0.3054	0.6305	ns
Muscle	L-FABPliv$^{-/-}$ vs. L-FABPint$^{-/-}$	-0.005246	-0.1308 to 0.1203	0.9878	ns

S-9. References used in Supporting Information

1. Pierri A, Krepich S. Per- and polyfluorinated alkyl substances (PFAS) from milk, eggs, butter, cheese, and fish using QuEChERS, SPE, and LC-MS/MS - phenomenex application note: TN-01234 2018 Available from: https://phenomenex.blob.core.windows.net/documents/a9406d77-c88e-49cb-9347-81efd828fc25.pdf.

2. Usui, K., Hayashizaki, Y., Hashiyada, M. and Funayama, M., 2012. Rapid drug extraction from human whole blood using a modified QuEChERS extraction method. Legal Medicine, 14(6), pp.286-296.
